# Traitement de la neuromyélite optique de Devic durant de la grossesse

**DOI:** 10.11604/pamj.2016.24.230.9167

**Published:** 2016-07-13

**Authors:** Moussa Toudou Daouda, Norlin Samuel Obenda, Hamid Assadeck, Diankanagbe Camara, Fatimata Hassane Djibo

**Affiliations:** 1 Service de Neurologie, CHU Hassan II, Fès, Maroc; 2 Service de Médecine et Spécialités Médicales, Hôpital National de Niamey, Niger; 3 Faculté des Sciences de la Santé, Université Abdou Moumouni de Niamey, Niger

**Keywords:** Neuromyélite optique et grossesse, corticothérapie, immunosuppresseurs, rituximab, immunoglobulines intraveineuses, plasmaphérèse, Neuromyelitis optica and pregnancy, corticosteroid therapy, immunosuppressive agents and rituximab, intravenous immunoglobulins, plasmapheresis

## Abstract

La neuromyélite optique de Devic est une pathologie inflammatoire démyélinisante du système nerveux central qui affecte électivement la moelle spinale, le nerf optique et les régions cérébrales à haute expression d’antigènes aquaporine 4. Il s’agit d’une pathologie auto-immune sévère due à des auto-anticorps dirigés contre l’aquaporine 4, à taux de morbidité et de mortalité élevé. Contrairement à d’autres pathologies inflammatoires notamment la sclérose en plaques ou polyarthrite rhumatoïde, la grossesse n’exerce aucune influence sur l’activité de la neuromyélite optique d’où la nécessité d’instaurer un traitement de fond durant toute la grossesse. La corticothérapie représente le traitement de premier choix de la neuromyélite optique durant la grossesse. D’autres traitements peuvent également être utilisés notamment le rituximab, certains immunosuppresseurs, les immunoglobulines. Le traitement par immunosuppresseurs ou rituximab est proposé lorsque la corticothérapie au long cours est contre-indiquée ou en cas d’inefficacité à celle-ci ou encore lorsque les effets secondaires sont intolérables. Les immunoglobulines sont administrées en cas de poussées sévères de la neuromyélite optique qui ne répondent pas aux bolus de methylprednisolone. Les immunoglobulines peuvent également être poursuivies seules à la dose 0,4g/kg/j toutes les 6 à 8 semaines jusqu’à l’accouchement. La plasmaphérèse est également une bonne alternative aux bolus de methylprednisolone lorsque les poussées sont très sévères.

## Abstract (en)

Neuromyelitis optica (Devic’s disease) is an inflammatory demyelinating disease of the central nervous system that mainly affects spinal cord, optic nerve and brain regions with high aquaporin 4 antigen expression. This is a severe autoimmune disease caused by autoantibodies directed against aquaporin 4 and associated with high morbidity and mortality. Unlike other inflammatory conditions such as multiple sclerosis or rheumatoid polyarthritis, pregnancy does not seem to influence the activity of neuromyelitis optica, hence the need for a thorough treatment during pregnancy. Corticosteroid therapy is the treatment of choice for neuromyelitis optica during pregnancy. Other treatments may also be used including rituximab, some immunosuppressive agents and immunoglobulins. Immunosuppressive treatment or rituximab is recommended when the long-term corticosteroid treatment is contraindicated, in case of inefficiency or if side effects are intolerable. Immunoglobulins are administered to patients with serious outbreaks of neuromyelitis optica which do not respond to bolus methylprednisolone. Immunoglobulins alone can also be continued at a dose of 0.4 g/kg/day for 6-8 weeks until delivery. Plasmapheresis is also a good alternative to bolus methylprednisolone when outbreaks are extremely severe.

## Introduction

Décrite pour la première fois en 1894 par Eugène Devic [1] et Fernand Gault [2], la neuromyélite optique de Devic (NMO) ou maladie de Devic est une pathologie inflammatoire démyélinisante sévère du système nerveux central (SNC) qui touche essentiellement le nerf optique et la moelle épinière [3]. Longtemps considérée comme une forme particulière de la sclérose en plaque (SEP), la NMO est de nos jours reconnue comme une entité à part, se différenciant de la SEP sur le plan clinique, épidémiologique, immunologique et anatomopathologique [3–5]. La découverte en 2004 de l´immunoglobuline G neuromyélite optique (NMO-IgG), un autoanticorps dirigé contre l’aquaporine 4 (AQP4) a permis la compréhension du mécanisme pathogénique de la NMO [6, 7]. La prévalence est plus élevée dans les populations non caucasiennes contrairement à la SEP [4, 8]. Le diagnostic repose sur la présence d’une névrite optique et d’une myélite aiguë transverse plus l’existence de deux des trois signes suivants: détection à l’imagerie par résonnance magnétique (IRM) d’une lésion de la moelle épinière couvrant au moins 3 vertèbres, l’IRM cérébrale initiale normale ou non caractéristique de sclérose en plaques, séropositivité aux NMO-IgG [9]. Le traitement doit être approprié et bien conduit en raison du taux de morbidité et de mortalité élevé de la maladie.

Si des études ont démontré que le risque de rechute pendant la grossesse est moindre dans certaines pathologies inflammatoires notamment la SEP, la polyarthrite rhumatoïde [10] ; aucune étude n’a signalé une modification de l’activité de la NMO pendant la grossesse en comparaison avec la période pré-grossesse, ainsi que pendant l’allaitement [11–13]. En outre, plusieurs cas de premier épisode de NMO survenue au cours de la grossesse ont été rapportés [14–17], étayant l’hypothèse de la non influence de la grossesse sur l’activité de la maladie. En l’absence de tout traitement durant la grossesse, le risque de poussées pouvant être graves et non réversibles est élevé ainsi que les fausses couches ou morts fœtales in utéro [10, 18]. A cet effet, un traitement de fond est indispensable dans le but de réduire au maximum l’activité de la maladie pendant la grossesse. Dans certains cas, malgré un traitement bien conduit, la maladie ne cesse de progresser. La corticothérapie par voie générale reste le traitement de première intention de la NMO au cours de la grossesse [13, 15]. D’autres traitements notamment le rituximab, certains immunosuppresseurs, les immunoglobulines, les échanges plastiques sont prescrits en cas d’absence de réponse thérapeutique à la corticothérapie ou en cas contre-indication à ce traitement [13, 15, 19–21]. Nous présentons, dans cette revue, les principaux traitements de la NMO au cours de la grossesse ainsi que leurs indications.

## Méthodes

Notre travail est une revue de la littérature. Nous avons effectué une recherche bibliographique sur PubMed, EMBASE, Scorpus et Sciencedirect, Google scholar, avec d’abord les mots clés ‘’Devic's neuromyelitis optica’’ ou ‘’ Devic’s neuro-optic myelitis’’ ou ‘’Neuromyelitis optica‘’ et ‘’pregnancy’’ puis avec les mots clés ‘’Devic's neuromyelitis optica’’ et ‘’treatment’’. A l’issue de notre recherche bibliographique, 38 articles ont été analysés et inclus cette revue de la littérature.

### Etat actuel des connaissances

#### Traitements

##### La corticothérapie

La corticothérapie par voie générale représente le traitement de premier choix de la NMO au cours de la grossesse [12–14, 20–22], plus précisément au premier trimestre de la grossesse. Les études de cas rapportés de patientes atteintes de NMO ayant reçu la corticothérapie pendant la grossesse, n’ont pas signalé d’effets tératogènes liés à ce traitement [14, 15, 17, 23, 24]. La corticothérapie s’est montrée efficace dans la plupart des cas, en permettant une stabilisation ou rémission de la maladie durant la grossesse [15, 16, 22, 23]. La méthode thérapeutique consiste en des perfusions intraveineuses de methylprednisolone à la dose 1g/jour pendant 3 à 5 jours [12, 13, 19, 22] voire 12 jours [25] en fonction de la réponse thérapeutique. Les bolus intraveineux de methylprednisolone sont, par la suite, relayés par une corticothérapie orale notamment la prednisone. La dose préconisée varie de 1 à 2 mg/kg/j pendant 6 à 8 semaines puis dégression progressive [14, 15]. La dose d’entretien pourrait varier de 20 à 40 mg/j. Des cas de chutes de la maladie étaient rapportés avec des doses de prednisone inférieures ou égales à 20 mg/j [13, 15, 18]. Dans d’autres situations, les bolus intraveineux de methylprednisolone peuvent être poursuivis seuls à la dose 1g/jour/mois jusqu’à l’accouchement [24], avec une meilleure tolérance clinique. L’usage au long cours des corticoïdes expose au risque de nombreux effets secondaires qui sont plus fréquents lorsque la dose administrée est élevée. Ainsi, il est important d’associer à la corticothérapie un traitement adjuvant lorsque la dose élevée. Ce traitement associera surtout une supplémentation vitaminocalcique et potassique dans le but de prévenir l’ostéoporose cortisonique et de compenser les fuites potassiques.

### Les échanges plasmatiques: plasmaphérèse

La plasmaphérèse est réalisée en l’alternative aux bolus intraveineux de methylprednisolone lorsque la réponse thérapeutique est insatisfaisante ou d’emblée réalisée lorsque les poussées sont très sévères [26, 27]. La réponse clinique à la plasmaphérèse est liée à l´élimination non spécifique de facteurs inflammatoires et humoraux notamment les IgG-NMO [28]. Le taux de réponse clinique à la plasmaphérèse varie de 45 à 74% [29]. En outre, La plasmaphérèse peut s’avérer efficace même plusieurs semaines après la poussée de NMO [26, 30, 31]. Le nombre de séances de plasmaphérèse est discuté cas par cas selon la sévérité de la poussée et la réponse thérapeutique. Le délai moyen de réponse thérapeutique, après les séances de plasmaphérèse, est très variable, de quelques jours à quelques semaines selon les patients et la sévérité de la poussée [29]. Après les échanges plasmatiques, un traitement d’entretien par corticothérapie orale ou immunosuppresseurs ou rituximab, doit être démarré pour prévenir les rechutes. La grossesse ne présente pas de contre-indication à la plasmaphérèse [15, 18, 23].

### Les immunoglobulines

Les immunoglobulines intraveineuses sont prescrites dans le traitement des attaques sévères de NMO qui ne répondent pas aux bolus intraveineux de methylprednisolone. Elles sont administrées à la dose de 0,4 g/kg/j pendant 5 jours consécutifs [32]. Elles permettent une amélioration clinique et une réduction significative du taux des rechutes [33]. Les perfusions intraveineuses d’immunoglobulines sont soit poursuivies à la dose 0,4 g/kg/j toutes les 6 à 8 semaines jusqu’à l’accouchement [32, 33], soit relayées par un autre traitement notamment corticothérapie orale ou immunosuppresseurs. Les immunoglobulines intraveineuses peuvent également être administrées en l’alternative à la plasmaphérèse et vice versa.

### Les immunosuppresseurs

L’azathioprine est l’immunosuppresseur le plus fréquemment utilisée pendant la grossesse. Les études des cas rapportés des patientes atteintes de NMO recevant ce traitement durant la grossesse n’ont pas signalé d’effets tératogènes [13, 34]. Chez l’homme, il n’existe pas dans la littérature de données d’études contrôlées montrant de malformations chez les enfants nés des femmes enceintes ayant bénéficié d’un traitement par azothioprine durant leur grossesse. Les immunosuppresseurs ont une efficacité très lente, s’observant au bout de 2 à 6 mois de traitement, d’où la nécessité d’introduire au début du traitement les bolus intraveineux de methylprednisolone toutes les 6 à 8 semaines pendant 6 mois. Le traitement par immunosuppresseurs nécessite une surveillance biologique à intervalles réguliers (chaque semaine le premier mois puis tous les 3 mois, comportant une numération formule sanguine et les transaminases) durant tout le traitement à cause leurs effets toxiques. Les immunosuppresseurs sont préférés aux corticoïdes en raison des effets secondaires de la corticothérapie au long cours qui sont parfois sévères. Ils sont prescrits lorsque la corticothérapie orale s’avère inefficace ou en cas de contre-indication à ce traitement. L’azathioprine est administrée à la dose de 100 à 150 mg/j [25]. Bien que ce traitement n’ait pas d’effets tératogènes prouvés, il devrait être évité surtout durant le premier trimestre de la grossesse. Lorsque la grossesse est survenue sous traitement par azathioprine ou encore lorsque ce traitement administré au premier trimestre de la grossesse, une surveillance obstétrique régulière peut s’avérer nécessaire surtout aux 8, 12 et 22 semaines d’aménorrhées.

### Le rituximab

Le rituximab est un anticorps monoclonal dirigé contre l’antigène CD20 des lymphocytes B [35], proposé comme traitement de fond après les bolus intraveineux de methylprednisolone ou comme traitement alternatif à la corticothérapie au long cours en cas de contre-indication à celle-ci ou de réponse thérapeutique insatisfaisante [20, 21]. La fixation du rituximab sur les antigènes CD20 induit la morte des lymphocytes B par apoptose et par conséquent entraine une diminution de la sécrétion des IgG-NMO [35, 36]. Le rituximab a montré son efficacité dans la prévention des rechutes de la NMO mais aussi il permet une stabilisation clinique de la maladie [37]. Il est administré à la dose de 1 g/j deux fois à intervalle de 2 semaines ou encore 375 mg/m2/semaine pendant 4 semaines [35–38]. L’évaluation clinique et du taux des lymphocytes CD20 sera pratiquée toutes les 6 semaines puis le rituximab sera réadministré, selon le même protocole, dès que le taux de CD20 est supérieur ou égal à 0,05% [37, 38]. Les études des cas rapportés n’ont pas signalé d’effets tératogènes lié à l’usage du rituximab chez les femmes enceintes [20, 21, 35]. Dans la littérature, il n’existe pas de données d’études contrôlées chez les femmes enceintes démontrant les effets tératogènes du rituximab. Cependant, une lymphopénie transitoire peut s’observer chez les enfants nés des patients ayant reçu le rituximab durant leur grossesse. L’inconvénient de l’usage du rituximab est l’exposition au risque infectieux pouvant être grave [37]. Ainsi, avant chaque cure du rituximab, il est formel de réaliser un bilan infectieux plus ou moins inflammatoire comportant une numération formule sanguine (NFS), une C-protein reactive (CRP), une étude cytobactériologique des urines, recherche de bacille de Koch (GèneXpert^®^, intradermoréaction à la tuberculine), sérologie hépatite B et C, une radiographie thoracique, électrophorèse des protéines sériques, vitesse de sédimentation. Bien qu’il n’existe pas d’études démontrant les effets tératogènes lié au traitement par rituximab, une surveillance obstétrique peut s’avérer nécessaire chez les femmes enceintes recevant ce traitement, surtout aux 8, 12 et 22 semaines d’aménorrhées.

### Indications thérapeutiques

Le traitement par corticothérapie, notamment les bolus intraveineux de methylprednisolone, est proposé en première intention en cas de poussées modérées de NMO. La corticothérapie peut être proposée seule comme traitement de fond de NMO durant toute la grossesse selon les détails ci-dessus (cf. à la corticothérapie). La plasmaphérèse est d’emblée proposée en cas de poussées sévères de NMO ou lorsque la réponse thérapeutique à la corticothérapie générale est très insatisfaisante. Les immunoglobulines peuvent être d’emblée proposées dans les poussées sévères ou en cas de réponse thérapeutique très peu satisfaisante à la corticothérapie générale ou à la plasmaphérèse. L’azathioprine est proposée en cas de contre-indication à la corticothérapie orale au long cours ou en cas de mauvaise tolérance à ce traitement. Le rituximab est proposé dans les formes sévères et réfractaires de NMO.

## Conclusion

La NMO est un syndrome associant une myélite et une névrite optique d’évolution et de sévérité variable. Comme des études ont démontré que la grossesse n’exerce aucune influence sur l’activité de la maladie, un traitement adéquat est indispensable durant toute la grossesse au vu de prévenir non seulement les rechutes mais aussi les fausses couches ou mort fœtale in utero. Des travaux ont montré que beaucoup de traitements pourraient être utilisés pendant la grossesse et qui sont sans effets tératogènes. Ces traitements sont proposés selon la sévérité de la maladie, de la réponse thérapeutique et de la tolérance. Dans certaines situations, le traitement pourrait être inefficace parce que le diagnostic est tardif et/ou la forme est d’emblée grave avec des lésions irréversibles.

### Etat des connaissances actuelle sur le sujet

- La grossesse n’exerce aucune influence sur l’activité de la NMO de Devic, d’où la nécessité d’instaurer un traitement de fond adéquat durant toute la grossesse;
- Taux de morbidité et de mortalité élevé de la maladie.

### Contribution de notre étude à la connaissance

- Les traitements susceptibles d’être utilisés durant la grossesse qui sont sans effets tératogènes;
- Contribution à l’amélioration de la prise en charge des femmes enceintes atteintes de la NMO.
